# Immune and inflammation: related factor alterations as biomarkers for predicting prognosis and responsiveness to PD-1 monoclonal antibodies in cervical cancer

**DOI:** 10.1007/s12672-022-00560-8

**Published:** 2022-09-28

**Authors:** Xihan Liu, Xi Zhang, Chang Liu, Wendi Mu, Jin Peng, Kun Song

**Affiliations:** 1 Department of Obstetrics and Gynecology, Qilu Hospital of Shandong University, Jinan, Shandong China; 2 Gynecologic Oncology Key Laboratory, Qilu Hospital of Shandong University, Jinan, Shandong China

**Keywords:** Cervical cancer, PD-1 monoclonal antibody, Tumor immunotherapy, Inflammation, Immune-related response gene signature

## Abstract

**Purpose:**

We aimed to elucidate the potential mechanisms of effective responsiveness to PD-1 monoclonal antibody and evaluate more reliable biomarkers to improve the ability to predict the populations of cervical cancer (CC) suitable for immunotherapy.

**Methods:**

Peripheral blood samples of CC patients undergoing anti-PD-1 therapy were collected before and after treatment. Differentially expressed genes (DEGs) were analyzed between partial response (PR) and progressive disease (PD) patients. A novel prognostic inflammation and immune–related response gene (IRRG) model was constructed and its prognostic role, correlation with tumor immunity and tumor mutation were evaluated.

**Results:**

DEGs in PR patient after treatment could predict the response to PD-1 monoclonal antibodies. Among PR-specific pathways, tumor immunity, leukocyte migration, and cytokine activities were prominently enriched. Additionally, an IRRG signature comprising CTLA4, AZU1, C5, LAT, CXCL2, GDF7, MPL, PPARG and CELA1 was established and validated to predict the prognosis of CC with great accuracy and specificity. This signature could reflect the tumor microenvironment (TME) and tumor mutational burden (TMB). We also found stimulated adaptive immunity and downregulated inflammation at baseline in patients with sensitive responses to PD-1 monoclonal antibody.

**Conclusion:**

We developed an IRRG signature and verified that it was an independent prognostic factor for predicting survival and could reflect a sensitive response to PD-1 monoclonal antibody, which plays a nonnegligible role in the TME of CC. Further investigations are warranted to confirm that patients with stimulated adaptive immunity and downregulated inflammation at baseline could achieve a better survival benefit from PD-1 monoclonal antibody.

**Supplementary Information:**

The online version contains supplementary material available at 10.1007/s12672-022-00560-8.

## Introduction

Cervical cancer (CC) is the second leading cause of cancer death in women, and the 5-year overall survival (OS) is only approximately 15% when it progresses to recurrence or metastasis [1, 2]. Programmed cell death-1 (PD-1) monoclonal antibody has been a promising therapeutic approach for CC [3–7]. It was first recommended by the National Comprehensive Cancer Network (NCCN) in 2018 for recurrent CC patients with “MSI-H/dMMR” subtype gene mutation [8], and has been suggested as a second-line therapy for patients with high PD-L1 expression in 2019. However, nearly 75% of patients do not effectively respond to anti-PD-1 therapy [9], and responses to pembrolizumab do not necessarily correlate with PD-L1 expression in CC study cohorts [10, 11], emphasizing the need for the stratification of patients who are likely to benefit from PD-1 monoclonal antibody.

Sensitive responses to anti-PD-1 therapy are dependent not only on the specific tumor properties but also on the activated immune system [12, 13]. To date, various predictive biomarkers, such as the frequencies of subsets of CD8^+^ T cells, CD4^+^ T cells, neutrophils and suppressive macrophages, have been discovered to be useful in monitoring the immune status of the host by examining cell compartments at tumor sites or in peripheral blood [14, 15]. Cytokines or chemokines, which are important immune regulators, were also identified as responder biomarkers [16]. However, the clinical usage of these biomarkers is far from fulfilled, and their predictive value is limited. This provided the rationale to evaluate more reliable biomarkers for the treatment response to immunotherapy.

Inflammation which is an emerging hallmark of cancer, is closely linked to tumorigenesis by interacting with all stages of cancer development through the inflammatory tumor microenvironment (TME) [17]. Several studies indicate that inflammation could predispose cells to the tumor generation via an inhibitory effect on antitumor immunity, changing the host immunity status toward a more tumor-promotive state by multiple signals and influencing immune, epithelial and cancer cells [18]. The interplay of inflammatory infiltration and pathways related to the immune response and tumorigenesis is complex and equivocal. On the one hand, activation of innate immune pathways is an essential precondition for the generation of adaptive immune responses, which is a prerequisite for effective immunotherapy responses and antitumor immune responses [19, 20]. On the other hand, the persistent existence of inflammatory substances will cause immunosuppression, which in turn promotes tumorigenesis [21].

In addition, an increasing number of studies have shown that inflammatory mediators play a pivotal role in both CC tumorigenesis and the immunotherapy response [22]. Thus, we focused on the potential of targeting inflammatory and immune pathways to shape the TME to favor checkpoint inhibition monotherapy. To date, several inflammatory and immune-based prognostic scores have been employed to predict prognosis and cancer immunotherapy efficacy in several cancers, such as the tumor inflammation signature (TIS) [23], the systematic immune-inflammation index (SII), and the panimmune-inflammation value (PIV) [24]. Nevertheless, the underlying molecular mechanisms that dictate the balance between the adaptive immune system and inflammatory factors within the TME remain unclear [25]. Precise, novel, and more predictive immune-inflammation molecular biomarkers have not yet been investigated thoroughly in CC patients.

In this study, we examined the gene expression of two kinds of patients (partial response (PR) and progressive disease (PD) patients) before and after treatment by blood-based tests in an attempt to determine the reasons for the different responses to the PD-1 monoclonal antibody, which is simple and patient-friendly. Here, we demonstrated that low baseline levels of inflammatory pathways such as the TNF, MAPK, NF-kappa B, IL-17 and HIPPO signaling pathways, and high levels of adaptive immune responses, e.g., T-cell activation and chemokine costimulation are predictors of sensitive responses to PD-1 monoclonal antibody. Additionally, an independent prognostic model based on an immune and inflammation-related response gene (IRRG) signature, which could reflect the TME of CC, was discovered and constructed by transcriptome analysis and The Cancer Genome Atlas (TCGA) database. A nomogram including the IRRG model was constructed to achieve better and more precise patient follow-up. Collectively, our findings discuss the clinical consideration of specific indications for PD-1 monoclonal antibody usage and demonstrate that this prognostic IRRG model plays essential roles in the stratification of patients likely to respond to anti-PD-1 monotherapy and in managing the prognosis of patients with CC.

## Materials and methods

### Sample collection

Patients diagnosed with CC who showed disease progression after chemotherapy and were treated with a PD-1 monoclonal antibody were included. All participants provided informed consent. The study followed the principles of the Declaration of Helsinki. The study was approved by the ethics committee of Qilu Hospital, Shandong University (Date: 2019/02/27, No. 2019004).

### Usage and dosage

Anti-PD-1 protein was administered intravenously every 2 weeks at a dose of 3 mg/kg.

### PD-L1 immunohistochemistry (IHC) staining

IHC staining was used to detect PD-L1 expression in formalin-fixed paraffin-embedded (FFPE) tumor tissue using E1L3N (AMoydx, Xiamen, CHN). PD-L1 expression was evaluated by the combined positive score (CPS), and a threshold of 1 was used to describe PD-L1 positivity (≥ 1) or negativity (0).

### Evaluation method

The target lesion was evaluated by spiral enhanced CT. Efficacy was assessed by using the Response Evaluation Criteria in Solid Tumors (RECIST version 1.1).

### Transcriptome analysis

Total RNA was isolated from patient peripheral blood samples using TRIzol reagent (Invitrogen) and then next-generation sequencing was performed by Novogene Co., Ltd. (Beijing, China). Differentially expressed genes (DEGs) between PR and PD patients prior to treatment initiation and after treatments were analyzed using edge R software. |Fold change|> 2 and p < 0.05 were used as the thresholds. Gene Ontology (GO) analysis and Kyoto Encyclopedia of Genes and Genomes (KEGG) pathway analysis were performed with OmicShare tools, a platform for data analysis (https://www.omicshare.com/tools). Enrichment analyses were performed using the Reactome dataset and DO dataset from OvoMagic (https://magic.novogene.com). The predicted interactions for the IRRGs were performed with the Search Tool for the Retrieval of Interacting Genes (STRING) database.

### Construction of the inflammation and immune—related PD-1 response gene signature and its clinical value

The Genotype-Tissue Expression (GTEx) and TCGA databases were used to download the data of normal cervix tissues and 306 CC patients’ transcriptome data with clinical information (Supplementary Table 1). Leas absolute shrinkage and selection operator (LASSO) regression was performed to identify an IRRG signature. The risk score was calculated for each sample according to the following formula: risk score $={\sum }_{i=1}^{n}\mathrm{Coef i}*\mathrm{Xi}$. Coef i is the coefficient of IRRGs and Xi is the gene expression value. Then, the patients were divided into high-risk score and low-risk score groups based on the best cutoff values and median cutoff values. Next, correlations of survival analysis, immune cell infiltration [26], and tumor mutational burden (TMB) [27] with the risk score were investigated. Finally, a nomogram was constructed on the basis of multivariate Cox regression analysis to ensure that this model was an independent prognostic factor for CC.

### RNA isolation and quantitative real-time PCR (qRT–PCR)

Total RNA was extracted from CC and normal cervical tissues from six patients with TRIzol reagent (Invitrogen). RNA was reverse transcribed with the HiScript II Q RT SuperMix for qPCR Kit (Vazyme). Then, qPCR was performed using SYBR Green mix (Vazyme) on a QuantStudio 3 system (Applied Biosystems, USA) to measure the expression levels of IRRGs. GAPDH was used as a control. The 2 ^− ΔΔCt^ method was used to quantify the qPCR data.

### Statistical analysis

SPSS Statistics (version 26) and Prism (version 9) were used to perform statistical analysis. Student’s *t* test was used to determine significance. R software packages (version 4.1.0) were used to analyze the clinical values of the IRRG model and to generate plots. The significance threshold was set at P-value < 0.05.

## Results

### Patient characteristics

**Fig. 1:**
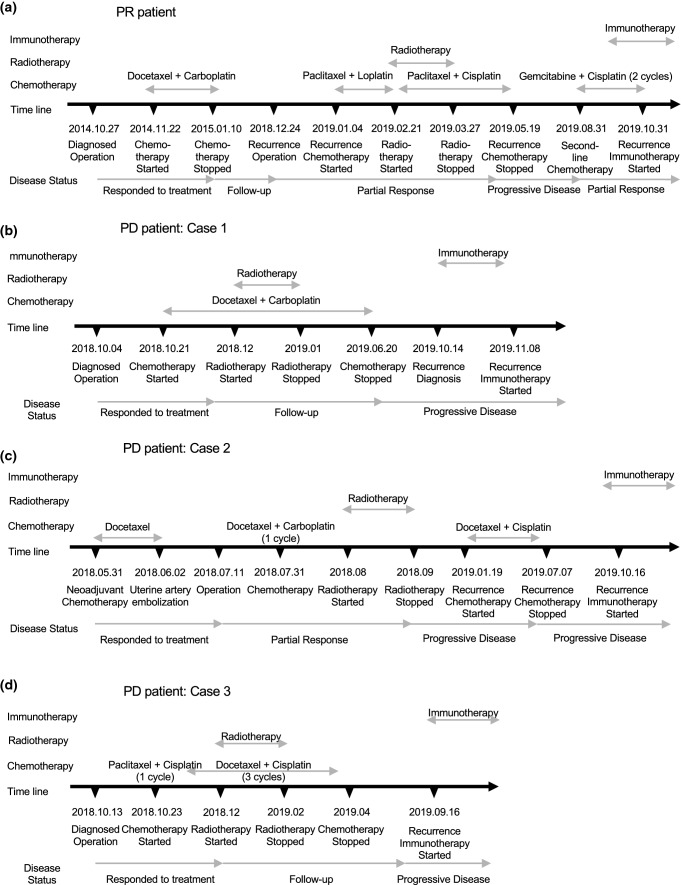
Timeline of patients’ receiving treatments and status. **A** PR patient receiving treatments and status before PD-1 monoclonal antibody therapy (Patient 4). **B**–**D** PD patient receiving treatments and disease status before PD-1 monoclonal antibody therapy (Patient 1–3)

The patient demographics and clinical outcomes following PD-1 monoclonal antibody treatment are summarized in Table 1. In total, four patients were included and had adequate tumor tissues available for PD-L1 staining. The staging of cancer was determined as International Federation of Gynecology and Obstetrics (FIGO) stage IV. All patients received type IV radical hysterectomy and pelvic lymphadenectomy, chemotherapy and radiotherapy but relapsed. These patients all received combined chemotherapy based on platinum and paclitaxel. One patient experienced metastasis and received combined chemotherapy including gemcitabine and cisplatin after metastasis. The patient treatment timeline is shown in Fig. 1.

**Table 1:** Patient and tumor characteristics, treatment and outcome

Characteristics of patients				
	No. of cases (PD1( +))			
	1	2	3	4
Age (years)	56	36	61	39
FIGO stage (2018)	IV	IV	IV	IV
Silva s	C	C	C	C
Histological grade	ADC, III	SCC, III	SCC, III	SCC, III
Tumor shape	Infiltrative	Infiltrative	Infiltrative	Infiltrative
Status of lymph node	Positive	Positive	Positive	Positive
Status of resection margins	Negative	Negative	Negative	Negative
Parametrium involvement	Yes	No	Yes	Yes
LVSI	Yes	Yes	Yes	Yes
Stromal invasion	Deep 1/3	Deep 1/3	Deep 1/3	Deep 1/3
Tumor size, cm	≥ 4	≥ 4	≥ 4	≥ 4
Operation method	Laparoscopy	Laparoscopy	Laparotomy	Laparotomy
Type of surgery	Type IV radical hysterectomy and pelvic lymphadenectomy	Type IV radical hysterectomy and pelvic lymphadenectomy	Type IV radical hysterectomy and pelvic lymphadenectomy	Type IV radical hysterectomy and pelvic lymphadenectomy
Clinical response	PD	PD	PD	PR
Adjuvant therapy				
CT cycle	5	8	4	11
RT cycle	28	1	25	25
RT Range	Pelvic	Pelvic	Pelvic	Pelvic
Comorbidity	Thrombosis history; hypertension	Renal insufficiency (stage I)	Diabetes; hypertension	Anemia (stage II)

*ADC* Adenocarcinoma; *SCC*: Squamous cell carcinoma; *FIGO* International Federation of Gynecology and Obstetrics; *Histological* *grade* I (well differentiated), II (moderately differentiated), III (poorly differentiated); *LVSI* lympho-vascular space invasion; *Stromal* *invasion* Superficial 1/3, Middle 1/3, Deep 1/3; *CT* chemotherapy; *RT* radiotherapy

### Patient outcomes following anti-PD-1 therapy

One patient (Patient 4) (one out of four patients, 25%) experienced a clinical benefit after 4 cycles of anti-PD-1 therapy with a 9-month progression-free survival (PFS) at the end point of observation. This patient had PR according to RECIST 1.1. In contrast, three patients (three out of four patients, 75%) had PD and did not experience a clinical benefit, which was confirmed with a scan after 3 months.

### Imaging changes after anti-PD-1 therapy

**Fig. 2:**
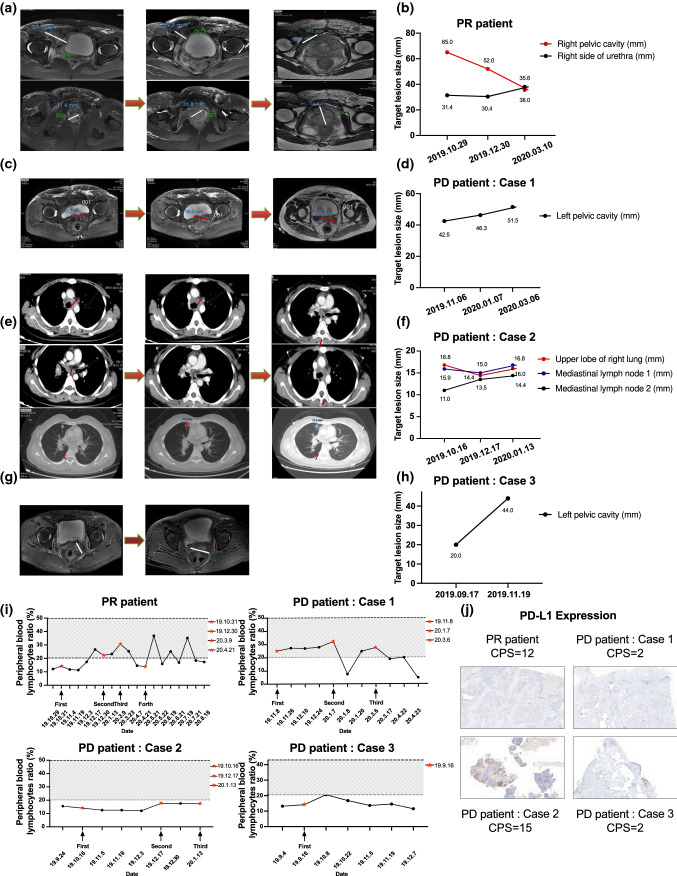
Detection of clinical indicators of patients. **A**–**H** Image changing trend of target lesion size of patients. **I** Change trend of peripheral blood lymphocyte ratio of patients. **J** PD-L1 expression of immunohistochemistry staining. The combined positive score (CPS) was used to represent the expression level

Representative radiologic responses after every cycle of anti-PD-1 therapy are shown in Fig. 2A–G. Of the 4 patients, 1 patient (patient 4, 25%) had PR with a target lesion in the right pelvic cavity shrinking from 65 to 38 mm. The sum of lesion diameters (SOD) value declined 15%, 30.9%, and 18.5% in each evaluation (Fig. 2A, B). Three patients (75%) had disease progression. Patient 1 had increased SOD values of 17.5% and 40% each (Fig. 2C, D). Patient 2 developed right lung metastasis, while the target tumor SOD decreased 1.77% and 2.2% (Fig. 2E, F). The target tumor SOD in patient 3 increased 31.25% 2 months later (Fig. 2G, H).

### Relationship between clinical features and response to anti-PD-1 monoclonal antibody

#### Hemogram analysis

It is vital to distinguish anti-PD-1 antibody responders from nonresponders by evaluating whether they are at the stage of reversible or irreversible CD8^+^ T-cell exhaustion by monitoring peripheral blood lymphocytes [28]. We investigated the correlation between putative circulating biomarkers and the response to anti-PD-1 antibody. Four patients underwent blood tests prior to treatment initiation and after treatment up to 5 to 10 months. The peripheral blood lymphocyte ratio was collected and evaluated. As shown in Fig. 2I, Patient 4 (PR patient) with a clinical response had increased peripheral blood lymphocytes every time after treatment except for the third treatment. In contrast, PD patients (Patients 1–3) showed no increase in the peripheral blood lymphocyte ratio, indicating that patients with PD might be at the stage of irreversible exhaustion and would not respond to anti-PD-1 antibody. This might be a reason why PR patient responded sensitively to anti-PD-1 antibody and obtained clinical benefits because of her activated immune system marked by systemically elevated peripheral blood lymphocytes, indicating that she has a strong lymphocyte sparing capacity.

#### PD-L1 assessment in tumors

Then, we analyzed PD-L1 expression in tumor samples using CPS. Surprisingly, patient outcomes were less correlated with PD-L1 expression. A CPS of 15 was observed in Patient 2, who had rapid symptomatic progression and symptomatic disease progression (Fig. 2J).

### Global changes in the transcriptome map of responders and nonresponders

We conducted whole blood RNA-seq for four patients (1 with clinical benefit, 3 without clinical benefit) at baseline and during treatment. The on-treatment samples were collected after 3 cycles of anti-PD-1 therapy.

**Fig. 3:**
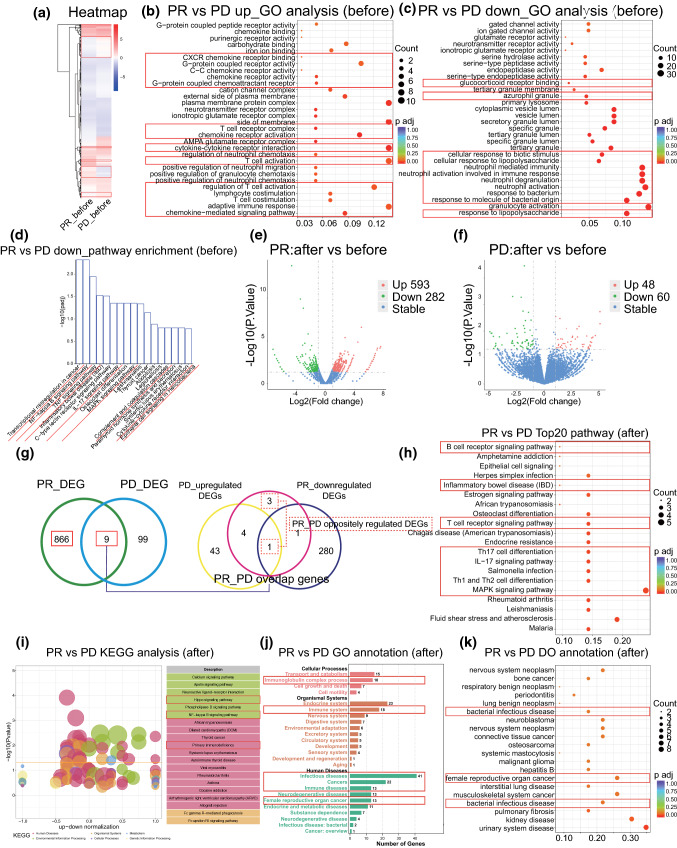
Global transcriptome changes between PR and PD patients before and after treatment. **A** Heatmap showing DEGs between PR and PD patients prior to PD-1 monoclonal antibody treatment. **B**, **C** GO analysis of upregulated (b) and downregulated (c) genes between PR and PD patients before treatment. **D** Pathway analysis of downregulated genes between PR and PD patients before treatment. **E–F** Volcano plots of DEGs in PR (e) and PD patients (f) after treatment. P < 0.05, log2FC > 1. **G** Venn diagram showing 866 PR patient’s special DEGs and 4 oppositely regulated DEGs in PR and PD patients after treatment, which add up to a changing-fate gene set. **H**, **I** Top 20 pathways and KEGG analysis of changing-fate gene set. **J**, **K** GO and DO analysis of changing-fate gene set

First, to discover potential critical biomarkers to predict the anti-PD-1 antibody response of patients without any treatment, we compared DEGs between PR and PD patients at baseline prior to anti-PD-1 therapy initiation (Fig. 3A, Supplementary Table 2). Interestingly, in PR patient, GO terms such as the adaptive immune response, lymphocyte and T-cell costimulation, and regulation of various immune cells, such as lymphocytes and granulocytes, were significantly activated (Fig. 3B). Neutrophil activation, response to bacteria and pathways, including the MAPK signaling pathway, NF-kappa B signaling pathway, TNF signaling pathway, IL-17 signaling pathway and transcriptional misregulation in cancer identified by GO and KEGG databases, were negatively regulated (Fig. 3C, D), indicating that the adaptive immune system was stimulated and inflammation was downregulated in PR patient compared with PD patients before treatment.

Next, we performed transcriptome sequencing of pretreatment and posttreatment peripheral blood samples obtained from the 4 patients to investigate the landscape of transcriptome alterations between patients with and without clinical benefit after treatment. A total of 593 upregulated genes and 282 downregulated genes were identified in PR patient after treatment (Fig. 3E, Supplementary Table 3), compared with 48 upregulated genes and 60 downregulated genes in PD patients (Fig. 3F, Supplementary Table 4). The number of alterable genes in PR patient was much greater than that in PD patients, indicating that PR patient may be more sensitive to PD-1 monoclonal treatment.

Then, we took the intersection of two changed gene sets in PR and PD patients to select PR patient-specific DEGs by excluding PD patient DEGs (Fig. 3G). A total of 866 genes were selected, together with 4 overlapping genes that were oppositely regulated in the two kinds of patients (1 downregulated gene in PR patient that was upregulated in PD patients, and 3 upregulated genes in PR patient that were downregulated in PD patients). We called these total 870 genes the changing fate gene set, which was assumed to perform antitumor functions and increase body sensitivity to anti-PD-1 therapy (Supplementary Table 5).

We conducted functional enrichment analysis of the changing-fate genes in PR patient. The analysis revealed that adaptive immune pathways and inflammation regulatory pathways were highly enriched. B and T cell receptor signaling pathway, Th17, Th1 and Th2 cell differentiation, the MAPK signaling pathway and the interleukin-17 signaling pathway were identified using the REACTOME database (Fig. 3H). The NF-kappa B and MAPK signaling pathways, which play critical roles in the inflammation process [29, 30], were identified by KEGG analysis (Fig. 3I). GO terms such as immune system, infectious diseases and cancers especially female reproductive organ cancer were highly enriched (Fig. 3J). DO analysis showed that the functions of the changing-fate genes were associated with female reproductive organ cancer progression, infectious disease initiation and tumor immunity (Fig. 3K). All DEGs that were divided into upregulated and downregulated groups with log fold change are separately exhibited in Supplementary Table 6 and 7.

### Establishment of a differentially expressed IRRG panel as a prognostic model

**Fig. 4:**
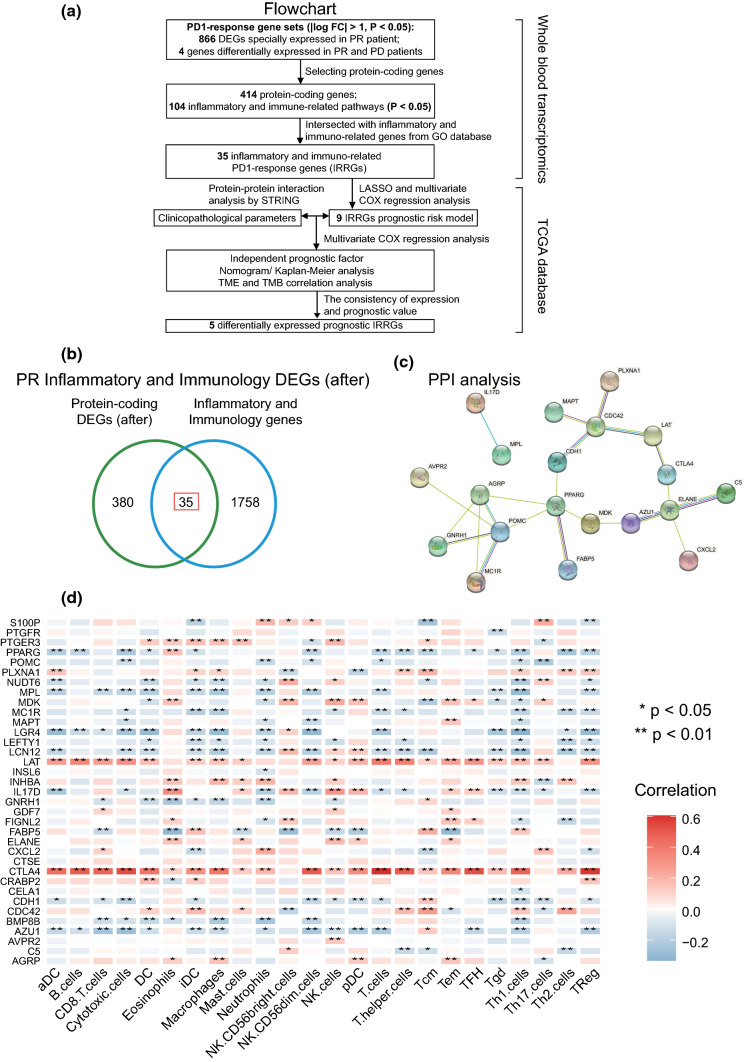
Establishment of an inflammatory and immune-related anti-PD-1 response gene (IRRG) panel. **A** Flow chart of IRRG selection and analysis. **B** Thirty-five IRRGs were intersected between 414 protein-coding DEGs in PR patient compared with PD patients after treatment and inflammatory and immune-related genes identified by GO database. **C** Protein–protein interaction analysis of 35 IRRGs by STRING database. **D** Correlation heatmap between IRRGs and immune cells. *P < 0.05, **P < 0.01, ***P < 0.001

Based on DEG functional analysis, we assumed that inflammatory and immune responses played an essential role in regulating effective responses to PD-1 monoclonal antibody treatment. Therefore, we sought to identify prognostic inflammatory and immune–related genes in CC. A flowchart was drawn to elaborate the gene selection progress more visually, as shown in Fig. 4A. Among the 414 protein-coding genes, we identified 104 immune-related pathways and 35 inflammatory and immune–related DEGs (Fig. 4A, B). Protein–protein interactions were generated by the STRING database to explore gene interactions (Fig. 4C). The correlation analysis between 35 inflammatory and immune–related DEGs and immune cell proportions was performed by the ssGSEA algorithm using the TCGA CC database [31, 32] (Fig. 4D). The results indicated that the upregulated DEGs such as CTLA4, LAT, INHBA, ELANE, CXCL2 and PTGER3, were significantly positively correlated with CD8 + T cells, cytotoxic cells, macrophages, dendritic cells, neutrophils and regulatory T cells, while the downregulated IRRGs, such as LEFTY1, FABP5, POMC, IL17D and GNRH1, were negatively correlated with those immune cells, indicating that the immune system of PR patient was significantly activated (Fig. 4D).

**Fig. 5:**
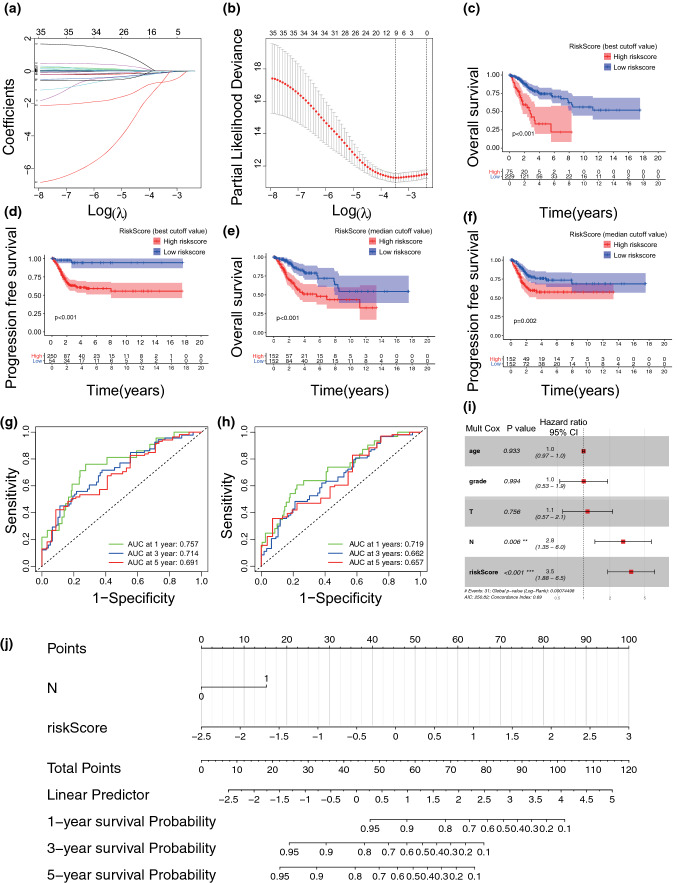
Identification of expression and prognostic value of IRRGs. **A**, **B** Heatmaps showing the expression of 35 IRRGs in PR and PD patient samples before and after treatment and in GSE173097 with normal tissues and primary cervical tumors. **C**, **D** Survival map of downregulated and upregulated IRRGs in PR patient after treatment across TCGA tumors. **E**, **F** Univariate and multivariate Cox regression of nine-candidate prognostic IRRG signature. **G**, **H** Kaplan–Meier (KM) analysis of prognostic factors for survival in TCGA CC database

Twenty-six of 35 inflammatory and immuno-related genes (74.29%) were upregulated in PR patient (Fig. 5A). We verified the expression levels of the inflammatory and immune–related DEGs between CC and normal cervical tissues in the GSE173097 dataset and across cancers in TCGA (Fig. 5B, C). We found that downregulated genes in PR patient, such as IL17D, MDK and GNRH1, which are known for their role in the innate immune system, are unfavorable prognostic factors highly expressed in primary cervical tumors and across cancers (Fig. 5C). Upregulated genes in PR patient such as AZU1, CDH1 and GDF7, are favorable factors expressed at low levels in CC samples in TCGA (Fig. 5D). Interestingly, we observed that CTLA4 was upregulated in patients with a clinical benefit, which is an inhibitory receptor for cytotoxic T lymphocyte (CTL) activation (Fig. 5D). We believe that these inflammatory and immune–related DEGs are the key regulators in PR patient who is sensitive to anti-PD-1 therapy. Univariate and multivariate Cox regression (Fig. 5E, F) and Kaplan–Meier analyses (Fig. 5G) were performed to identify prognostic genes.

**Fig. 6:**
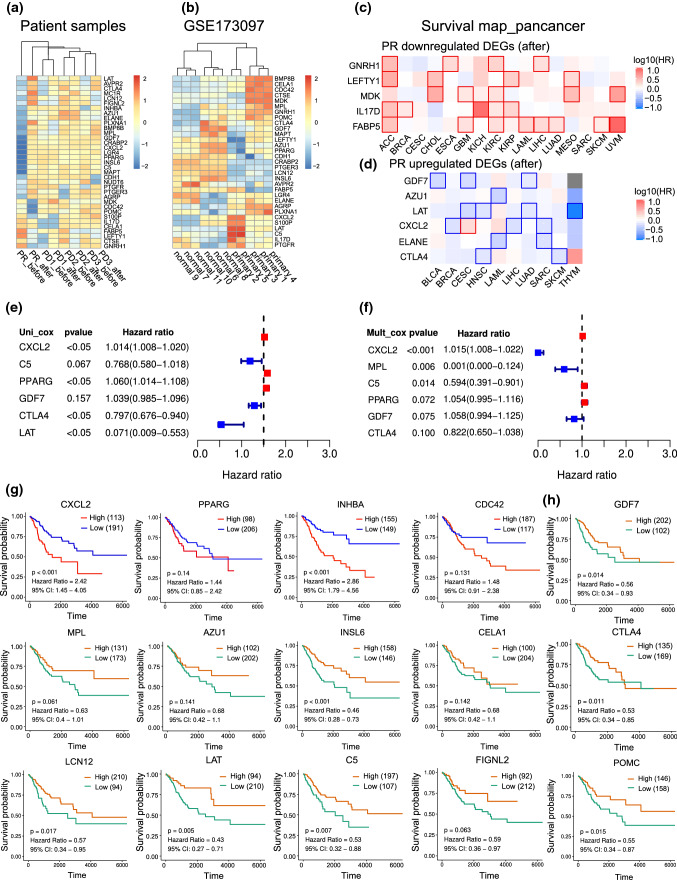
Construction of IRRG model and its predictive value for OS. **A** The LASSO was used to validate IRRG model parameter selection. **B** The LASSO coefficient map of IRRG model. **C**, **D** KM curves of overall survival (OS) and progression free survival (PFS) for CC patients divided by the best cutoff values from TCGA database. **E**, **F** KM curves of OS and PFS for CC patients divided by the median values from TCGA database. **G**, **H** Receiver operating characteristic (ROC) curves of IRRG model for OS and PFS prediction. **I** Multivariate Cox analysis of age, grade, tumor, metastasis and IRRG model with OS. **J** Construction of nomogram including nodal metastasis and risk score to predict CC patient OS

Then, we applied LASSO Cox regression to construct an IRRG model using TCGA database (Fig. 6A, B). Finally, nine candidates, CTLA4, AZU1, C5, LAT, CXCL2, GDF7, MPL, PPARG, and CELA1, were identified. We further calculated the risk score according to the following formula: risk score $={\sum }_{i=1}^{n}\mathrm{Coef i}*\mathrm{Xi}$ (Table 2). The patients were divided into high-risk and low-risk groups by the median and best cutoff values. The high-risk group had worse OS and PFS rates than the low-risk group with the best and median cutoff values (Fig. 6C–F), with 64.4% and 71.0% 5-year OS rates and 72.9% and 71.0% 5-year PFS rates in the high-risk group compared with 84.6% and 88.1% and 96.2% and 82.8% in the low-risk group (Table 2). In addition, the negative predictive value (NPV) of the IRRG model for PFS was 96%, and the positive predictive value (PPV) for OS was 36% (Table 2). The specificity and sensitivity of the IRRG signature in predicting 1-, 3-, and 5-year OS and PFS for CC patients were validated by the area under the curve (AUC) value. (Fig. 6E, F). Overall, the IRRG model is a promising prognostic signature for CC patients.

**Table 2:** Clinical significance of the 9-IRRGs signature-based risk score in predicting prognosis of patients in cervical cancer

Index	Cutoff	High risk cases	Low risk cases	5-Year survival rate		P-value	PPV%	NPV%
				High risk	Low risk			
OS	Median	152	152	0.710	0.881	p < 0.001	29	12
PFS	Median	152	152	0.710	0.828	p = 0.002	29	17
OS	Best	76	228	0.644	0.846	p < 0.001	36	15
PFS	Best	251	53	0.729	0.962	p < 0.001	27	96

*OS* overall survival; *PFS* progression-free survival; *PPV* positive predictive value; *NPV* negative predictive value

The best cutoff of survival analysis was calculated by R package

### The IRRG model was an independent prognostic factor with potential clinical value

To validate whether the IRRG model was an independent predictor of OS, we first performed multivariate Cox regression with age, grade, tumor, nodal metastasis and IRRG model risk score. The results showed that nodal metastasis (P = 0.006) and high-risk score (P < 0.001) were independent prognostic factors (Fig. 6G). Then a nomogram was constructed with these two independent prognostic factors (C-index = 0.688), which had moderate accuracy in predicting patients’ 1-,3-, and 5-year survival probabilities and served as a clinical prognostic approach for CC patients.

### The IRRG model was associated with the TME

**Fig. 7:**
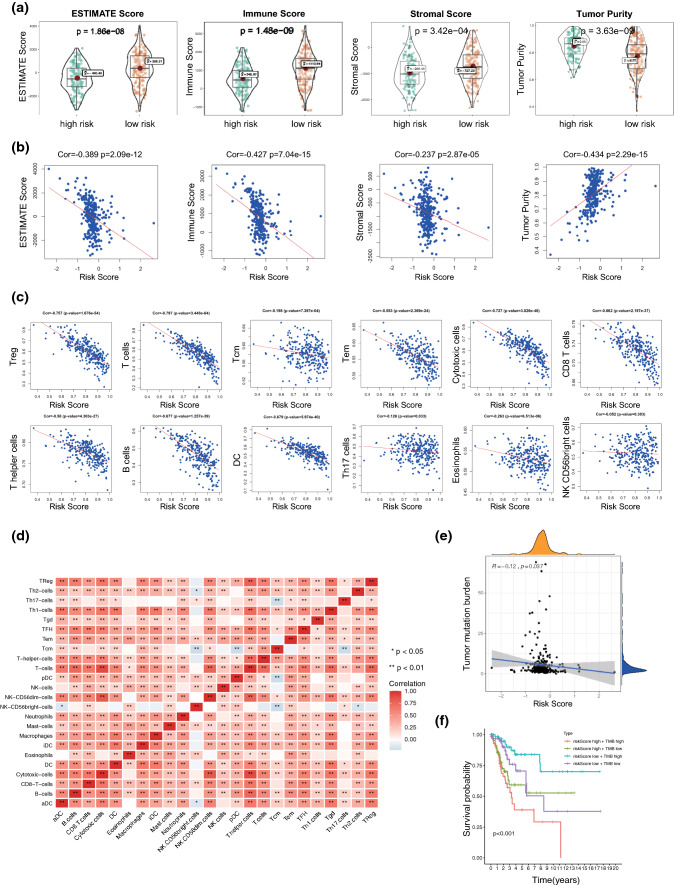
The correlation of IRRG model between tumor immune landscapes and TMB. **A** The level of ESTIMATE score, immune score, stromal score and tumor purity in high-risk and low-risk score groups. **B** The correlation analysis of IRRG model and ESTIMATE score, immune score, stromal scores and tumor purity. **C** Correlation analysis of IRRG model and the levels of 24 subtypes of immune cells evaluated by the ssGSEA algorithm. **D** Correlation analysis of immune cells. **E** The correlation between IRRG model and tumor mutation burden (TMB). **F** KM analysis of patients with risk score and TMB

We then wanted to determine the correlation of our model and the TME to determine whether the IRRG model could reflect the TME to some extent. As a result, a high-risk score was associated with lower ESTIMATE, immune, stromal scores and higher tumor purity with P < 0.05 (Fig. 7A). Correlation analysis was further conducted between the risk score and TME. There was a significant negative correlation between the risk score and TME (Fig. 7B). Immune cells play pivotal roles in the TME, and different proportions may reflect different clinicopathological characteristics [33]. Thus, we performed correlation analysis between the IRRG model and immune cell proportions with ssGSEA. Subtypes of T cells including helper T cells, regulatory T cells, cytotoxic cells, CD8 T cells, Tcm cells and Tem cells, were negatively correlated with the IRRG model, while Th17 cells, eosinophils and NK CD56bright cells seemed to have a weak relationship with the risk score (Fig. 7C). The correlations between immune cells are shown in Fig. 7D. NK-CD56bright cells were negatively correlated with some infiltrated immune cells, including activated Th2 cells, Tcm cells, and dendritic cells (Fig. 7D).

### The IRRG model is associated with TMB

TMB is another important biomarker for an effective immunotherapy response. We observed that the risk score was weakly negatively associated with TMB (P = 0.037, R = − 0.12) (Fig. 7E). However, no obvious significant difference in TMB was observed between the two risk score clusters (Fig. S1A) and survival analysis revealed that CC patient prognosis was not related to TMB (Fig. S1B, C). Nevertheless, interestingly, bivariate analysis combining the risk score and TMB showed that patients with high TMB and high-risk scores had the poorest prognosis (Fig. 7F).

### Validation of the gene panel expression in CC by qPCR and RNA-seq

**Fig. 8:**
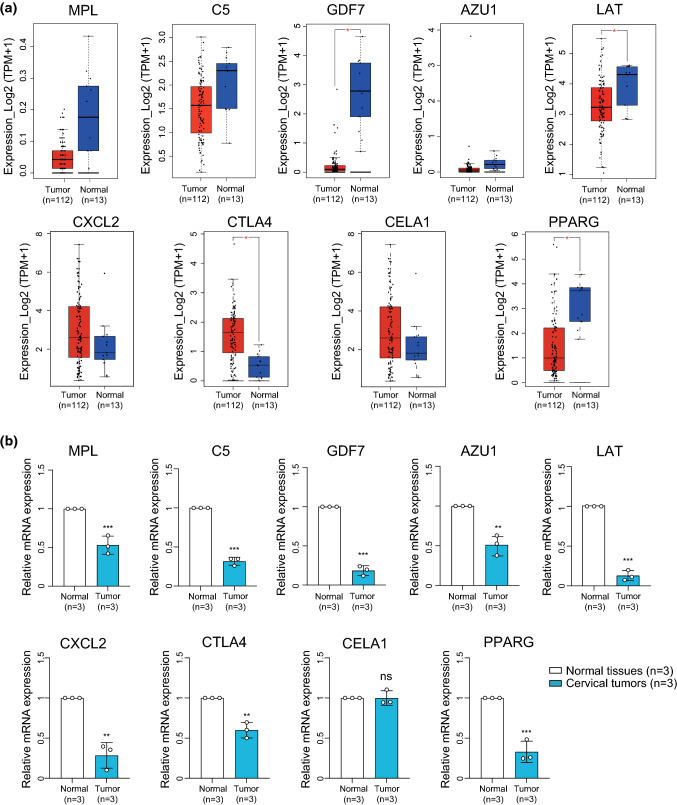
Validation of the IRRG panel mRNA expression in CC and normal cervical tissues by public database and qPCR. **A** The mRNA expression levels of nine signature IRRGs identified in PR patient after treatment by RNA-seq were verified in CC tissues (n = 112) and normal cervical tissues (n = 13) in TCGA database. **B** The mRNA expression levels of nine IRRGs in CC (n = 3) and normal cervical tissues (n = 3) by qPCR. *P < 0.05, **P < 0.01, ***P < 0.001

At the end of the study, we verified IRRG expression both in the TCGA CC database and in our Qilu CC tissue samples. MPL, C5, GDF7, AZU1, LAT and PPARG were expressed at low levels in CC tissues compared with normal tissues at the mRNA level (Fig. 8A). We also observed consistent significant expression differences in these IRRGs in our Qilu cohort, including 6 patients with CC and normal tissues. However, CTLA4, CXCL2 and CELA1 showed slightly different tendencies in the two databases, indicating that more samples need to be enrolled to perform verification (Fig. 8B).

## Discussion

The prognosis of patients with recurrent cervical cancer remains dismal due to less precise strategies and ineffective prognostic biomarkers for screening specific patients [34]. Immunotherapy is approved for CC patients who are positive for PD-L1 or who have the “MSI-H/dMMR” subtype gene mutation [35]. However, whether the indications are suitable and effective requires discussion. In our study, the enrolled patients all positively expressed PD-L1. Nevertheless, we found that not all PD-L1-positive patients responded to anti-PD-1 therapy. Three patients whose PD-L1 CPSs were 15, 2, and 2 had PD. Only 1 patient whose CPS was 12 achieved PR. Hence, there is an unmet clinical need to identify effective biomarkers for anti-PD-1 application to improve patient outcomes in treating CC.

Blood-based testing is a promising approach for earlier diagnosis and prognostic analysis and continuous monitoring, which could replace more invasive tissue sampling and examinations. Analyzing RNA from whole blood samples is technically rather simple, yet very informative. A recent study demonstrated that the whole blood transcriptome predicts gene expression for other tissues in the body [36]. The expression level of many transcripts in blood cells are also responsive to their micro-environment [37]. In order to find biomarkers for these purposes and identify the underlying mechanisms of effective response to PD-1 antibody therapy, we compared the biological condition and signs of PR and PD patients at baseline and after treatments using their whole blood RNA-seq data.

Growing evidence indicates that an activated immune system is the cornerstone for effective immunotherapy responses [38–40]. While inflammatory response is a crucial driver of immunotherapy resistance [41]. Importantly, in our study, vibrant and mobilized immune-related pathway function and low pretreatment levels of inflammation markers were identified in PR patient at baseline. The inflammation-related signaling pathways such as NF-kB, TNF, MAPK and IL-17 signaling pathways were identified downregulated in PR patient. Furthermore, GO and KEGG pathways such as recruitment of T-cell subsets and leukocyte migration were significantly found, which could indicate that PR patient had less inflammation and a better immunity status, so she might have a stronger immune system function to react to PD-1 monoclonal antibody treatment.

Inflammation participates in tumorigenesis and predisposes patients to all stages of cancer development [42]. Studies have elucidated that inflammatory mediators are often associated with multiple tumor properties [18]. Previous reports have found that the induction of inflammation, such as the nuclear factor-kappa B (NF-kB)-activated signaling pathway, results in tumor progression and metastasis [43, 44]. Similarly, IL-17 is another signaling pathway triggered by inflammatory cytokines that can promote tumor proliferation [45].

Intriguingly, we compared the DEGs in PR patient after anti-PD-1 monotherapy to those in PD patients and found that those changing-fate DEGs were highly enriched in the immune-related pathways and inflammatory processes annotated by GO and KEGG databases, with nearly 10% of them being inflammatory and immune-related genes. This phenomenon suggested that boosting the immune system and lowering the risk of inflammatory responses might be important prerequisites for an effective response to PD-1 antibody treatment. This is the reason that we emphasized understanding the role of inflammatory and immune pathways in response to immunotherapies.

In recent years, in-depth studies have developed a mass of immune-related prognostic signatures to predict immunotherapy response and have investigated the relationship between immune landscape and prognosis in CC [46–50]. For instance, Yu et al. [51] established an immune infiltration-based signature and demonstrated that a better prognosis in CC patients seemed to be relatively immune-inflamed with greater immune cell infiltration, which was consistent with our findings. Li et al. [33] established a classification of immune’s subtypes of CC predicting prognosis and immunotherapy responses. However, the association of immune infiltration and inflammation was not definitively pinpointed; and the signatures were based on bioinformatics analysis of public data and lacked recruited verification cohorts for predicting immunotherapy response. Here, for the first time, to delineate vital markers and signals that affect inflammation and immunity during tumor development and immunotherapy efficacy, we identified and established an independent inflammatory and immune–related prognostic model derived from our own clinical whole blood transcriptome for CC patients. Due to the relative scarcity of appropriate whole blood transcriptome verification datasets of CC patients thus far; and it has been shown that the whole blood transcriptome possesses > 80% expression similarity to other tissues of the human body [37]; the changes in the expression levels of individual genes reflect alterations in the environment of whole blood and may also reflect organ-specific changes [37]; additionally, extending whole blood transcriptomics to tissues seems extremely promising, considering that blood might reflect tumor tissue transcriptional changes to some extent. Hence, we used the TCGA CC tumor tissue database for IRRG model verification to obtain a comprehensive characterization of the whole blood and tumor tissue transcriptomic signature. We evaluated the clinical outcomes, TME and TMB of CC patients using this model. A high risk score was significantly associated with poor prognosis. In addition, patients with high risk scores exhibited lower ESTIMATE, immune and stromal scores, higher tumor purity and no significant relevance to TMB. High enrichment of tumor-infiltrating lymphocytes (TILs) in the TME has previously been found in the remaining tumor and is strongly correlated with tumor progression, aggressiveness, and sensitive responses to immunotherapy [39, 52]. We found IRRG model risk score negatively correlated with multiple subtypes of T cells such as including helper T cells, regulatory T cells, cytotoxic cells, CD8 T cells, Tcm cells and Tem cells, suggesting a close linkage between IRRG model and TME.

The nine prognostic IRRGs perform vital functions and cover many aspects of inflammation and immune processes. For example, LAT and CTLA4 are associated with T-cell activation and suppression [53, 54]. ELANE and AZU1 regulate antimicrobial activity and monocyte chemotaxis and act as important mediators of inflammation [55, 56]. MPL may regulate TPO-R-dependent immunological responses as a receptor for thrombopoietin [57]. GDF7 may serve as a ligand of the TGF-β superfamily. C5, which encodes a component of the complement system and has previously been shown to be significantly differentially expressed in anti-PD-1 treatment-resistant and -sensitive melanoma patients [58], was upregulated in PR patient. CXCL2 is a chemokine that exhibits an antimicrobial function as part of secreted proteins in immune and inflammatory processes [59], and it was also upregulated in PR patient. The expression of these nine candidate genes was verified by qPCR, transcriptome analysis from our own dataset and the TCGA CC database.

However, human cancers show great heterogeneity based on different genetic backgrounds, environments, habits, microbes and virus proportions [18]. Our limitations are that few patients were enrolled in this study to eliminate individual differences, and the study lacked another independent whole blood transcriptome dataset to verify the IRRGs as sensitive biomarkers for the selection of patients with an effective anti-PD-1 response. In the coming future, we will collect more clinical specimens to better confirm our conclusion.

## Conclusion

As inflammation and antitumor immunity play instrumental roles in tumorigenesis [42], it is imperative to discover essential immune and inflammatory pathways in immunotherapy resistance. In conclusion, we explored inflammatory and immune pathways and identified an independent prognostic IRRG model to predict survival and the response to PD-1 monoclonal antibody treatment in patients with CC, which could also reflect immune infiltration status. Our study sheds light on the potential of combinational biomarkers for immunotherapy to improve anti-PD-1 therapeutic efficacy. Future research is undoubtedly needed to uncover the molecular mechanisms of immunity and protumor inflammation, especially in increasing the effectiveness of immunotherapy.

## Supplementary Information

Additional file 1

Additional file 2

Additional file 3

Additional file 4

Additional file 5

Additional file 6

Additional file 7

Additional file 8

## Data Availability

Raw counts for RNA-seq transcriptome data and corresponding clinical data are available for download from the Gene Expression Omnibus (GEO) repository (GSE205247).

## Code availability

The codes generated for this study are available on request to the corresponding author.

## References

[1] Kumar SK, Callander NS, Alsina M, Atanackovic D, Biermann JS, Chandler JC (2017). Clinical practice guidelines in oncology. J Natl Compr Canc Netw.

[2] Guitarte C, Alagkiozidis I, Mize B, Stevens E, Salame G, Lee Y-C (2014). Glassy cell carcinoma of the cervix: a systematic review and meta-analysis. Gynecol Oncol.

[3] Liu Y, Wu L, Tong R, Yang F, Yin L, Li M (2019). PD-1/PD-L1 inhibitors in cervical cancer. Front Pharmacol.

[4] Duranti S, Pietragalla A, Daniele G, Nero C, Ciccarone F, Scambia G (2021). Role of immune checkpoint inhibitors in cervical cancer: from preclinical to clinical data. Cancers.

[5] Verhoeven Y, Quatannens D, Trinh XB, Wouters A, Smits ELJ, Lardon F (2021). Targeting the PD-1 axis with pembrolizumab for recurrent or metastatic cancer of the uterine cervix: a brief update. Int J Mol Sci.

[6] Sherer MV, Kotha NV, Williamson C, Mayadev J (2022). Advances in immunotherapy for cervical cancer: recent developments and future directions. Int J Gynecol Cancer.

[7] Dyer BA, Zamarin D, Eskandar RN, Mayadev JM (2019). Role of immunotherapy in the management of locally advanced and recurrent/metastatic cervical cancer. J Natl Compr Canc Netw.

[8] Bradley K, Frederick P, Reynolds RK, Tanner E, Comprehensive RHL, urban R. Cervical Cancer. 2022.

[9] Budhwani M, Turrell G, Yu M, Frazer IH, Mehdi AM, Chandra J (2021). Immune-inhibitory gene expression is positively correlated with overall immune activity and predicts increased survival probability of cervical and head and neck cancer patients. Front Mol Biosci.

[10] Frenel J-S, Le Tourneau C, O’Neil BH, Ott PA, Piha-Paul SA, Gomez-Roca CA (2016). Pembrolizumab in patients with advanced cervical squamous cell cancer: preliminary results from the phase Ib KEYNOTE-028 study. J Clin Oncol.

[11] De Felice F, Marchetti C, Palaia I, Ostuni R, Muzii L, Tombolini V (2018). Immune check-point in cervical cancer. Crit Rev Oncol Hematol.

[12] Le DT, Durham JN, Smith KN, Wang H, Bartlett BR, Aulakh LK (2017). Mismatch repair deficiency predicts response of solid tumors to PD-1 blockade. Science.

[13] Walsh RJ, Tan DSP (2021). The role of immunotherapy in the treatment of advanced cervical cancer: current status and future perspectives. J Clin Med.

[14] Heeren AM, Rotman J, Stam AGM, Pocorni N, Gassama AA, Samuels S (2019). Efficacy of PD-1 blockade in cervical cancer is related to a CD8+FoxP3+CD25+ T-cell subset with operational effector functions despite high immune checkpoint levels. J Immunother cancer.

[15] de Vos van Steenwijk PJ, Ramwadhdoebe TH, Goedemans R, Doorduijn EM, van Ham JJ, Gorter A (2013). Tumor-infiltrating CD14-positive myeloid cells and CD8-positive T-cells prolong survival in patients with cervical carcinoma: clinical benefit of CD14+ cells in cervical cancer. Int J Cancer.

[16] Mitsuhashi A, Okuma Y (2018). Perspective on immune oncology with liquid biopsy, peripheral blood mononuclear cells, and microbiome with non-invasive biomarkers in cancer patients. Clin Transl Oncol.

[17] Hanahan D, Weinberg RA (2011). Hallmarks of cancer: the next generation. Cell.

[18] Greten FR, Grivennikov SI (2019). Inflammation and cancer: triggers, mechanisms, and consequences. Immunity.

[19] Woo S, Fuertes MB, Corrales L, Spranger S, Michael J, Leung MYK (2015). STING-dependent cytosolic DNA. Trends Immunol.

[20] Pedersen JG, Madsen AT, Gammelgaard KR, Aggerholm-Pedersen N, Sørensen BS, Øllegaard TH (2020). Inflammatory cytokines and ctdna are biomarkers for progression in advanced-stage melanoma patients receiving checkpoint inhibitors. Cancers.

[21] Misra S, Hascall VC, Markwald RR, O’Brien PE, Ghatak S (2002). Inflammation and cancer. Nature.

[22] Ott PA, Bang Y-J, Piha-Paul SA, Razak ARA, Bennouna J, Soria J-C (2019). T-cell–inflamed gene-expression profile, programmed death ligand 1 expression, and tumor mutational burden predict efficacy in patients treated with pembrolizumab across 20 cancers: KEYNOTE-028. J Clin Oncol.

[23] Damotte D, Warren S, Arrondeau J, Boudou-Rouquette P, Mansuet-Lupo A, Biton J (2019). The tumor inflammation signature (TIS) is associated with anti-PD-1 treatment benefit in the CERTIM pan-cancer cohort. J Transl Med.

[24] Gambichler T, Said S, Abu Rached N, Scheel CH, Susok L, Stranzenbach R (2022). Pan-immune-inflammation value independently predicts disease recurrence in patients with Merkel cell carcinoma. J Cancer Res Clin Oncol.

[25] Shalapour S, Karin M (2019). Pas de Deux: control of anti-tumor immunity by cancer-associated inflammation. Immunity.

[26] Yoshihara K, Shahmoradgoli M, Martínez E, Vegesna R, Kim H, Torres-Garcia W (2013). Inferring tumour purity and stromal and immune cell admixture from expression data. Nat Commun.

[27] Jiang H, Zheng Y, Qian J, Mao C, Xu X, Li N (2021). Efficacy and safety of sintilimab in combination with chemotherapy in previously untreated advanced or metastatic nonsquamous or squamous NSCLC: two cohorts of an open-label, phase 1b study. Cancer Immunol Immunother.

[28] Hatae R, Chamoto K, Kim YH, Sonomura K, Taneishi K, Kawaguchi S (2020). Combination of host immune metabolic biomarkers for the PD-1 blockade cancer immunotherapy. JCI Insight.

[29] Kyriakis JM, Avruch J (2012). Mammalian MAPK signal transduction pathways activated by stress and inflammation: a 10-year update. Physiol Rev.

[30] Zinatizadeh MR, Schock B, Chalbatani GM, Zarandi PK, Jalali SA, Miri SR (2021). The Nuclear Factor Kappa B (NF-kB) signaling in cancer development and immune diseases. Genes Dis.

[31] Barbie DA, Tamayo P, Boehm JS, Kim SY, Moody SE, Dunn IF (2009). Systematic RNA interference reveals that oncogenic KRAS-driven cancers require TBK1. Nature.

[32] Bindea G, Mlecnik B, Tosolini M, Kirilovsky A, Waldner M, Obenauf AC (2013). Spatiotemporal dynamics of intratumoral immune cells reveal the immune landscape in human cancer. Immunity.

[33] Li Y, Lu S, Wang S, Peng X, Lang J (2021). Identification of immune subtypes of cervical squamous cell carcinoma predicting prognosis and immunotherapy responses. J Transl Med.

[34] Jiang P, Zou L, Wei L, Cheng G, Sun B, Zhang F (2021). Chinese expert consensus on iodine125 seed implantation for recurrent cervical cancer in 2021. Front Oncol.

[35] Seebacher NA, Stacy AE, Porter GM, Merlot AM (2019). Clinical development of targeted and immune based anti-cancer therapies. J Exp Clin Cancer Res.

[36] Basu M, Wang K, Ruppin E, Hannenhalli S (2021). Predicting tissue-specific gene expression from whole blood transcriptome. Sci Adv.

[37] Liew C-C, Ma J, Tang H-C, Zheng R, Dempsey AA (2006). The peripheral blood transcriptome dynamically reflects system wide biology: a potential diagnostic tool. J Lab Clin Med.

[38] Sharma P, Allison JP (2015). The future of immune checkpoint therapy. Science.

[39] Paijens ST, Vledder A, de Bruyn M, Nijman HW (2021). Tumor-infiltrating lymphocytes in the immunotherapy era. Cell Mol Immunol.

[40] Kim SI, Cassella CR, Byrne KT (2021). Tumor burden and immunotherapy: impact on immune infiltration and therapeutic outcomes. Front Immunol.

[41] Jinushi M, Chiba S, Yoshiyama H, Masutomi K, Dosaka-akita H, Yagita H (2014). All use subject to JSTOR terms and Conditions tumor-associated macrophages regulate tumorigenicity and anticancer drug responses of cancer stem/initiating cells. Proc Natl Acad Sci USA.

[42] Grivennikov SI, Greten FR, Karin M (2010). Immunity, inflammation, and cancer. Cell.

[43] Elyada E, Pribluda A, Goldstein RE, Morgenstern Y, Brachya G, Cojocaru G (2011). CKIα ablation highlights a critical role for p53 in invasiveness control. Nature.

[44] Pribluda A, Elyada E, Wiener Z, Hamza H, Goldstein RE, Biton M (2013). A Senescence-inflammatory switch from cancer-inhibitory to cancer-promoting mechanism. Cancer Cell.

[45] Sahu U, Biswas D, Prajapati VK, Singh AK, Samant M, Khare P (2021). Interleukin-17—a multifaceted cytokine in viral infections. J Cell Physiol.

[46] Yang S, Wu Y, Deng Y, Zhou L, Yang P, Zheng Y (2019). Identification of a prognostic immune signature for cervical cancer to predict survival and response to immune checkpoint inhibitors. OncoImmunology.

[47] Zhang W (2020). Prognostic implications of immune-related genes’ (IRGs) signature models in cervical cancer and endometrial cancer. Front Genet.

[48] Yao H, Jiang X, Fu H, Yang Y, Jin Q, Zhang W (2022). Exploration of the immune-related long noncoding RNA prognostic signature and inflammatory microenvironment for cervical cancer. Front Pharmacol.

[49] Mei J, Xing Y, Lv J, Gu D, Pan J, Zhang Y (2020). Construction of an immune-related gene signature for prediction of prognosis in patients with cervical cancer. Int Immunopharmacol.

[50] Qu X, Shi Z, Guo J, Guo C, Qiu J, Hua K (2021). Identification of a novel six-gene signature with potential prognostic and therapeutic value in cervical cancer. Cancer Med.

[51] Yu S, Li X, Zhang J, Wu S (2021). Development of a novel immune infiltration-based gene signature to predict prognosis and immunotherapy response of patients with cervical cancer. Front Immunol.

[52] Petitprez F, Meylan M, de Reyniès A, Sautès-Fridman C, Fridman WH (2020). The tumor microenvironment in the response to immune checkpoint blockade therapies. Front Immunol.

[53] Dinur-Schejter Y, Zaidman I, Mor-Shaked H, Stepensky P (2021). The clinical aspect of adaptor molecules in T cell signaling: lessons learnt from inborn errors of immunity. Front Immunol.

[54] Rowshanravan B, Halliday N, Sansom DM (2018). CTLA-4: a moving target in immunotherapy. Blood.

[55] Neuenfeldt F, Schumacher JC, Grieshaber-Bouyer R, Habicht J, Schröder-Braunstein J, Gauss A (2022). Inflammation induces pro-NETotic neutrophils via TNFR2 signaling. Cell Rep.

[56] Daigo K, Yamaguchi N, Kawamura T, Matsubara K, Jiang S, Ohashi R (2012). The proteomic profile of circulating pentraxin 3 (PTX3) complex in sepsis demonstrates the interaction with azurocidin 1 and other components of neutrophil extracellular traps. Mol Cell Proteomics.

[57] Re A, Cora D, Puliti AM, Caselle M, Sbrana I (2006). Correlated fragile site expression allows the identification of candidate fragile genes involved in immunity and associated with carcinogenesis. BMC Bioinformatics.

[58] Weber JS, Sznol M, Sullivan RJ, Blackmon S, Boland G, Kluger HM (2018). A serum protein signature associated with outcome after anti-PD-1 therapy in metastatic melanoma. Cancer Immunol Res.

[59] De Filippo K, Dudeck A, Hasenberg M, Nye E, van Rooijen N, Hartmann K (2013). Mast cell and macrophage chemokines CXCL1/CXCL2 control the early stage of neutrophil recruitment during tissue inflammation. Blood.

